# Delayed diagnosis of lung cancer due to misdiagnosis as worsening of sarcoidosis: a case report

**DOI:** 10.1186/s12890-020-1105-2

**Published:** 2020-03-21

**Authors:** Hong-Joon Shin, Min-Seok Kim, Bo Gun Kho, Ha Young Park, Tae-Ok Kim, Cheol-Kyu Park, In-Jae Oh, Yu-Il Kim, Young-Chul Kim, Yoo-Duk Choi, Sung-Chul Lim

**Affiliations:** 1 Department of Internal Medicine, Chonnam National University Medical School, Gwangju, Republic of Korea; 2 Department of Pathology, Chonnam National University Medical School, Gwangju, Republic of Korea

**Keywords:** Sarcoidosis, Lung cancer, Concurrent, Lymphadenopathy

## Abstract

**Background:**

The concurrence of sarcoidosis and primary lung cancer is very rare. We report a very rare case with a delayed diagnosis of primary lung cancer due to its misdiagnosis as worsening of pulmonary sarcoidosis.

**Case presentation:**

A 68-year-old man presented to the outpatient department for evaluation of a mass in the right hilar area with lymphadenopathies in subcarinal and both interlobar areas on chest computed tomography (CT). Sufficient core samples were obtained from subcarinal and bilateral interlobar lymph nodes using endobronchial ultrasonography (EBUS) guided transbronchial needle aspiration (TBNA). EBUS could not reach the right hilar lymph node due to its high angle. The pathologic findings were consistent with sarcoidosis. After 5 months, chest CT revealed aggravation of the right upper paratracheal lymphadenopathy. Assuming worsening of sarcoidosis, he was prescribed an oral corticosteroid for 5 months. However, follow-up chest CT showed a newly developed right lower paratracheal lymphadenopathy and worsening right hilar lymphadenopathy. Bronchoscopy and EBUS were performed once again. Transbronchial lung biopsy from the right upper lobe and EBUS-TBNA from the right lower paratracheal lymph node revealed adenocarcinoma from the lung.

**Conclusions:**

Although coexistence of sarcoidosis and lung cancer is very rare, the clinician should consider the possibility of accompanying lung cancer in sarcoidosis patients who are not responding to initial corticosteroid therapy.

## Background

Sarcoidosis is characterized by noncaseating granulomatous inflammation involving multiple organs [1–3]. Lung cancer should be excluded to confirm a diagnosis of sarcoidosis [4], although the concurrence of sarcoidosis and lung cancer has been reported [5–8]. Herein, we report a very rare case of pulmonary sarcoidosis co-existing with lung cancer, with delayed diagnosis of primary lung cancer due to misdiagnosis as worsening of pulmonary sarcoidosis.

## Case presentation

A 68-year-old man presented to the outpatient department (OPD) with chest computed tomography (CT) scan abnormality. He was an ex-smoker with 4.5 pack-years. His past medical history indicated that he had been taking medication for hypertension, but did not disclose history of diabetes mellitus or other cardiovascular disorders. A chest CT scan was performed at a local clinic before the patient presented to our OPD. He was suspected to have primary lung malignancy with metastasis to the lymph nodes.

His physical examination was unremarkable. Complete blood count was normal at 11,900/mm^3^, and C-reactive protein level, electrolyte panel, liver function studies, renal function tests, and coagulation profile were all within normal limits. Electrocardiography revealed normal sinus rhythm.

**Fig. 1:**
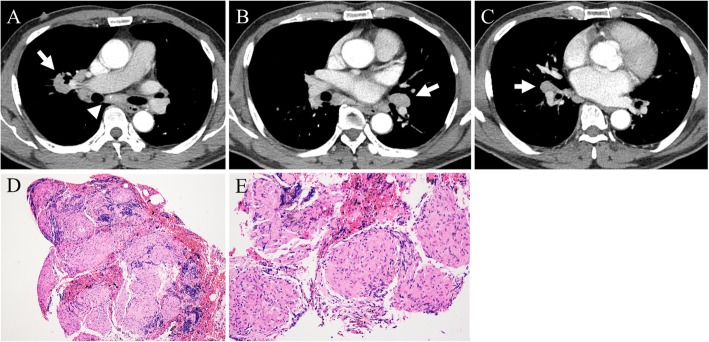
Chest CT scans at presentation and histopathological findings from first EBUS-TBNA. **a** Subcarinal lymphadenopathy (arrow head) and right hilar lymphadenopathy (arrow). **b** Left interlobar lymphadenopathy (arrow). **c** Right interlobar lymphadenopathy (arrow). **d** 100× field of H&E stain (hematoxylin and eosin stain) with noncaseating granuloma. **e** 200× field of H&E stain with noncaseating granuloma

**Fig. 2:**
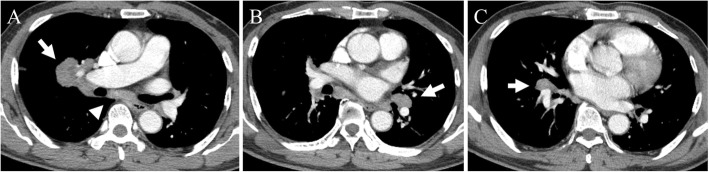
Chest CT scans at 5 months after diagnosis of sarcoidosis. **a** Aggravating right hilar lymphadenopathy (arrow) and no interval change of subcarinal lymphadenopathy (arrow head). **b** No interval changes of left interlobar lymphadenopathy (arrow). **c** No interval changes of right interlobar lymphadenopathy (arrow)

**Fig. 3:**
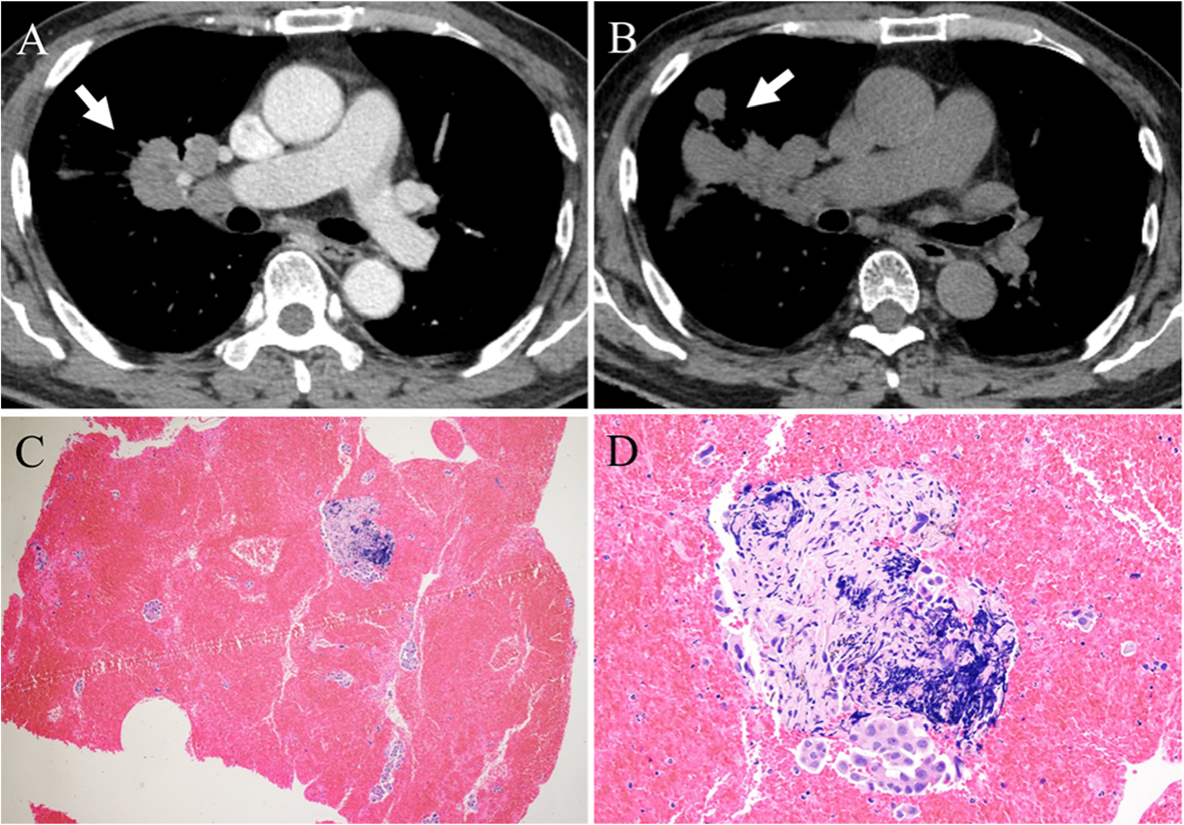
Chest CT scans after treatment with systemic corticosteroids and histopathological findings from second EBUS-TBNA. **a** Slightly aggravating right hilar lymphadenopathy after 3 months of 30 mg oral prednisone daily (arrow). **b** Markedly aggravating right hilar lymphadenopathy after an additional 2 months of 40 mg oral prednisone daily (arrow). **c** 20× field of H&E stain indicating adenocarcinoma, **d** 200× field of H&E stain indicating adenocarcinoma

He was suspected to have primary lung cancer with metastasis to the intrathoracic lymph nodes. Lymphadenopathies were observed in the subcarinal, right hilar and bilateral interlobar areas (Fig. 1a-c) on the chest CT scan performed at the local clinic. Because bronchoscopic findings were non-specific, an endobronchial ultrasonography (EBUS) was performed. Sufficient core samples were obtained from the subcarinal and bilateral interlobar lymph nodes using EBUS guided transbronchial needle aspiration (TBNA). Sampling at the right hilar lymph node was not possible via EBUS-TBNA as the position of the right hilar lymph node was too high for EBUS to reach. The pathologic findings showed noncaseating granulomas without malignant cells, consistent with sarcoidosis (Fig. 1d, e). As the patient intended to manage his pulmonary sarcoidosis at the local clinic, no further follow-up took place at the OPD. Five months later, however, he presented again to our OPD with aggravating right hilar lymphadenopathy (Fig. 2). The patient had not been treated with corticosteroid since the diagnosis of pulmonary sarcoidosis. Assuming worsening of sarcoidosis, he was prescribed 30 mg per day oral prednisone for 3 months. At 3 months follow-up, chest CT revealed waxing and waning of the right hilar lymphadenopathy (Fig. 3a). We decided to increase the dose of oral prednisone to 40 mg per day. However, at 2 months follow-up, the chest CT scan showed a newly developed right lower paratracheal lymphadenopathy and worsening right hilar lymphadenopathy, despite improvement of subcarinal and both interlobar lymphadenopathies (Fig. 3b). Fiberoptic bronchoscopy and EBUS were performed again. There was no endobronchial mass or destructive mucosal lesion on bronchoscopy. Transbronchial lung biopsy from the right upper lobe and EBUS-TBNA from the right lower paratracheal lymph node were performed. The final pathologic results revealed adenocarcinoma from the lung (Fig. 3c, d). He was transferred to a cancer center for treatment of lung cancer.

## Discussion

We describe a rare case of a delayed diagnosis of lung cancer due to misdiagnosis as worsening of pulmonary sarcoidosis. Sarcoidosis is a multi-systemic disease, which most commonly involves the lung, up to 95% of the time [1]. Diagnosis of sarcoidosis is confirmed by tissue biopsy with the exclusion of other possible diseases such as tuberculosis, lymphoma, and lung cancer [4]. Recently, EBUS guided TBNA has been frequently used to perform tissue biopsy from intrathoracic lymphadenopathies. However, it may be challenge to biopsy all intrathoracic lymph nodes through EBUS guided TBNA, because certain lymph nodes are difficult to access with EBUS.

Lung cancer must be excluded to confirm the diagnosis of pulmonary sarcoidosis [4]. There have been many reports of pulmonary sarcoidosis misdiagnosed as lung cancer [9–11]. While rare, the coexistence of pulmonary sarcoidosis and lung cancer has been reported [5–8]. The relationship between sarcoidosis and cancer is unclear, although three hypotheses have been suggested. First, persistent sarcoidosis may increase the risk of development of cancer [12–14]. Second, sarcoid-like reactions may develop in patients with cancer as a result of an immune response associated with cancer antigen or other factors [15–17]. Third, sarcoidosis and cancer may develop simultaneously without any relationship [18, 19]. In this case, lung cancer may have been present in the right hilar area at the time of sarcoidosis diagnosis. However, since sarcoid-like reactions and sarcoidosis cannot be distinguished pathologically, it is unclear whether the tissues obtained from EBUS-TBNA were sarcoidosis or sarcoid-like reactions.

Performing EBUS-TBNA is preferable over surgical procedure in patients with mediastinal and hilar lymphadenopathies [20, 21], and is an essential procedure to diagnose sarcoidosis [22]. The diagnostic yield of EBUS-guided TBNA is as high as 86 to 91.4% in patients with pulmonary sarcoidosis [20, 23]. Although Ernst et al. reported the diagnostic yield of EBUS-TBNA to be 91% in patients with single hilar lymph node [24], access to hilar lymph nodes may be more difficult compared to other intrathoracic lymph nodes. There are few studies on biopsies of hilar lymph nodes in the literature evaluating the efficacy of EBUS-TBNA compared to other intrathoracic lymph nodes [21, 25, 26]. In this case, while EBUS-TBNA was possible in the subcarinal and both interlobar lymph nodes, it was impossible to perform EBUS-TBNA at the right hilar lymph node because of its high angular position.

Systemic corticosteroid administration has been accepted as an initial treatment for sarcoidosis, and typically consists of 20–40 mg prednisone daily for 6–12 weeks [2, 3]. If there is no response to the initial corticosteroid treatment or development of toxic effects, immunosuppressant drugs can be added [2, 3]. In this case, there was no response to 30 mg prednisone daily after 3 months and deciding whether to increase dose of corticosteroids, add immunosuppressant drugs, or re-biopsy was a challenge. Ultimately, it was decided to increase prednisone to 40 mg daily for 2 months, and the diagnosis of lung cancer was delayed. Although positron emission tomography (PET) scan was not performed in this case, a PET scan would have been a good option if it were performed at the time of no response to initial corticosteroid treatment. As lung cancer has been reported to have a higher standardized uptake value of fluorodeoxyglucose than sarcoidosis, PET scan could be a good tool for distinguishing lung cancer from sarcoidosis [27].

## Conclusion

Further evaluation of the association between sarcoidosis and cancer is warranted. Although coexistence of sarcoidosis and lung cancer is very rare, clinicians should fully examine the possibility of accompanying lung cancer in sarcoidosis patients who are not responding to initial corticosteroid therapy.

## Data Availability

Data sharing is not applicable to this article as no datasets or analyzed during the current study.

## Abbreviations

CT: Computed tomography
EBUS: Endobronchial ultrasonography
OPD: Outpatient department
PET: Positron emission tomography
TBNA: Transbronchial needle aspiration
